# Achieving efficient red room-temperature phosphorescence in two-dimensional hybrid halide perovskites by manganese doping

**DOI:** 10.3389/fchem.2025.1533513

**Published:** 2025-03-21

**Authors:** Hui Zhu, Suqin Wang, Ming Sheng, Shao Bo, Yu He, Zhuang Liu, Min Li, Guangtao Zhou

**Affiliations:** ^1^ College of Engineering, Shandong Xiehe University, Jinan, China; ^2^ School of Materials Science and Engineering, Shandong University, Jinan, China; ^3^ Key Laboratory for Organic Electronics and Information Displays and Jiangsu Key Laboratory for Biosensors, Institute of Advanced Materials (IAM), Nanjing University of Posts and Telecommunications, Nanjing, China

**Keywords:** room-temperature phosphorescence, two-dimensional, halide perovskites, manganese doping, energy transfer

## Abstract

Red room-temperature phosphorescence (RTP), characterized by its long emission wavelength and lifetime, has broad applications in various fields, including bioimaging, light-emitting diodes (LEDs), information encryption, and photodetectors. Two-dimensional (2D) hybrid halide perovskites are a novel class of organic-inorganic hybrid composite materials. Their diverse binary combinations offer a versatile platform for designing energy transfer pathway and introducing emerging optical phenomena, making them promising candidates for developing the next-generation of RTP materials. However, currently reported red RTP halide perovskites are scarce and often suffer from low quantum efficiency and short lifetime. Herein, we developed a series of 2D hybrid halide perovskites with efficient red RTP through doping manganese ions and found that the modified materials exhibit a significantly enhanced photoluminescence quantum yield of 66.38%, which is eight times higher than that of the original perovskite host. Moreover, the phosphorescence lifetime can be extended up to 12 ms. In-depth mechanistic investigations reveal that the exceptional luminescent performance originates from efficient energy transfer be-tween the host matrix and manganese ions. Furthermore, red-emitting LEDs have been successfully fabricated with these RTP materials as emitting layers, highlighting the promising applications of 2D hybrid halide perovskites in lighting applications.

## 1 Introduction

Red room-temperature phosphorescence (RTP), with long emission wavelength and extended lifetime, holds significant potential for a wide range of applications, including bioimaging, light-emitting diodes (LEDs), information encryption, and photodetectors (Zhao et al., 2023; Deng et al., 2023; Fateminia et al., 2017; Xu et al., 2023; Wang et al., 2019a; Wang et al., 2019b; Zhu et al., 2022; Zhao et al., 2020a). Two-dimensional (2D) hybrid halide perovskites are a novel type of organic-inorganic hybrid material, where the integration of organic and inorganic components at the molecular level provides an effective platform for energy transfer (Su et al., 2022; Li et al., 2020; Gong et al., 2018; Gao et al., 2021; Zhang et al., 2019; Peng et al., 2017; Hsu et al., 2021; Liu et al., 2022; Liu et al., 2023). The emission spectra of 2D perovskites can be systematically tuned through employing different types of organic chains to modulate the radiative recombination pathway (Deng et al., 2023; Zhao et al., 2020b; Ma et al., 2019). This unique 2D structure allows for the generation of strong and persistent RTP, making 2D hybrid halide perovskites promising candidates for the development of next-generation RTP materials. Additionally, the rigid inorganic layers in 2D hybrid halide perovskites can enhance the phosphorescence of the organic components by controlling the energy transfer from Wannier excitons to triplet excitons (Ema et al., 2008). In a previous report, Lam et al. successfully integrated organic luminophores into 2D hybrid halide perovskites, achieving red RTP through energy transfer from the inorganic layer to the organic triplet state (Hu et al., 2018). However, the resulting RTP exhibited a short lifetime of just 2.1 ms and a low photoluminescence quantum yield (PLQY) of only 3%. Therefore, although red RTP has been demonstrated in 2D hybrid halide perovskites, challenges such as low PLQY remain, highlighting the need for further development of efficient red RTP materials in 2D hybrid halide perovskites systems.

Compared with the noble metals, manganese element with an atomic number of 25 has abundant reserves, is environmentally friendly and inexpensive. Manganese ion (Mn^2+^) dopants, having a 3 d^5^ electron configuration, can introduce a new emission center in semiconductor luminescent materials, endowing them promising photoluminescence properties, such as a wide color tuning range, reversible color change, reduced self-absorption etc. Specially, Mn^2+^ ion has been widely utilized as a dopant to induce red emission in various halide perovskites (Zhang et al., 2020; Han et al., 2022; Locardi et al., 2018; Das Adhikari et al., 2017; Sun et al., 2019; Li et al., 2019; Wang et al., 2022; He et al., 2022; Chen Y. et al., 2022). For example, Wang et al. successfully synthesized zero-dimensional (0D) (ABI)_4_MnBr_6_ halide perovskites (ABI = 2-aminobenzimidazole), which exhibits red light emission with a peak wavelength at 629 nm and a PLQY of 80% (Yan et al., 2021). The strong red emission is attributed to the d-d orbital transition (^4^T_1_→^6^A_1_) of Mn^2+^ coordinated in an octahedral configuration within the 0D perovskite structure. Moreover, Zhang et al. reported the hydrothermal synthesis of a crystal by doping Mn^2+^ into another 0D double perovskite Cs_2_NaInCl_6_ (Chen X. et al., 2022). This transparent crystal exhibits red emission at 610 nm under ultraviolet (UV) excitation and sustains luminescence for up to 2,500 s after the excitation is turned off. In addition, Han et al. developed a Mn^2+^-doped CsCdCl_3_ perovskite crystal, which demonstrates robust luminescence and RTP properties even at high temperatures (423 K) and in humid environments (He et al., 2022). The concentration of Mn^2+^ not only regulates the duration of luminescence but also enhances the PLQY from 37.1% to 91.4%. In another study, Kundu et al. synthesized 2D Mn^2+^-doped (C_4_H_9_NH_3_)_2_PbBr_4_ perovskite material, and in-depth mechanism exploration confirms efficient energy transfer from host excitons to Mn^2+^, resulting in intense orange-yellow luminescence with a maximum PLQY of 37% (Biswas et al., 2017). Aforementioned findings indicate that Mn^2+^ serves as a unique luminescent center, and doping with Mn^2+^ not only induces new optical phenomena but also imparts additional properties to materials, such as enhanced stability and quantum efficiency.

In this work, a series of 2D hybrid halide perovskite luminescent material were synthesized by doping Mn^2+^ into 2D hybrid halide perovskites (C_8_H_9_NO_2_)_2_CdCl_4_, abbreviated as P-MACC. The electron-donating alkoxy groups on the benzene ring were designed to fine-tune the electron density and shift their triplet levels. As expected, the resulted materials P-MACC:x%Mn, where x represents the theoretical molar doping percentage of Mn^2+^, exhibit strong red phosphorescence emission at 625 nm, attributed to the ^4^T_1_→^6^A_1_ transition of Mn^2+^. At room temperature, P-MACC:20% Mn demonstrates a maximum PLQY of 66.89%, which is eight times higher than that of P-MACC (8.69%), with Mn^2+^ ions lifetime of 12 ms. Time-resolved photoluminescence spectra reveal an efficient energy transfer from the triplet states of P-MACC to the dopant Mn^2+^, facilitating the high-efficiency red RTP. Subsequently, based on the superior luminescent properties of P-MACC:20% Mn, electroluminescent LEDs were assembled to highlight their potential as highly efficient luminescent materials for illumination applications.

## 2 Materials and methods

### 2.1 Materials

3,4-(methylenedioxy)benzylamine or so-called piperonylamine (P-MA, 97%), cadmium chloride (CdCl_2_, 99%), manganese chloride (MnCl_2_, 99%) were purchased from Aladdin. HCl (37 wt%, in water), ethanol and acetone were purchased from Nanjing Chemical Reagent Co. Ltd. All reagents and solvents were used without further purifications. Inspired by reported results, we selected P-MA as organic components in our work because the electron-donating alkoxy groups on the benzene ring have been designed to fine-tune the electron density and shift their triplet levels (Hu et al., 2018; Wu et al., 2022; Hu et al., 2019).

### 2.2 Synthesis of organic amines salts P-MACl

P-MACl. As shown in Supplementary Figure S1, under an ice-water bath, 8 mL of ethanol was placed in a 25 mL round-bottom flask. To this system, 1.0 mmol (125 μL) of P-MA was added, followed by the dropwise addition of 3 mL of HCl with continuous stirring. As the solution was stirred, the product P-MACl began to precipitate out. After allowing the reaction to proceed for 1 h, the product was collected. The product was then subjected to vacuum filtration using a filtration apparatus. During the filtration process, the products were washed six times with acetone. The washed products were stored in a centrifuge tube, sealed with parafilm, and placed in a vacuum drying oven for further characterizations. The final yield product in this synthesis procedure was 0.1633 g (approximately 87%).

### 2.3 Synthesis of 2D hybrid halide perovskites

P-MACC. As shown in Supplementary Figure S2, under ambient conditions, P-MACl (0.8 mmol, 0.1505 g) and CdCl_2_ (0.4 mmol, 0.0733 g) were placed in a 25 mL round-bottom flask. To this mixture, 5 mL of ethanol was added, and the system was thoroughly shaken. The mixture was then heated to 90°C and stirred continuously at this temperature for 1 h until a clear solution was obtained. The clear solution was then transferred to a clean sample vial. After allowing the vial to stand at room temperature for a period of time, the product P-MACC began to precipitate out. The product was then collected through vacuum filtration, washed 6 to 7 times with acetone, and stored in a centrifuge tube, sealed with parafilm. Finally, the product was placed in a vacuum drying oven for further characterizations. The final yield product in this synthesis procedure was 0.1899 g (approximately 85%).

P-MACC:x%Mn. As shown in Supplementary Figure S3, the synthesis of P-MACC:x%Mn were carried out in a similar way to that of P-MACC by replacing CdCl_2_ with a certain amount of MnCl_2_. The final yield rates in this synthesis are higher than 80% for all products.

### 2.4 Characterizations

Crystalline structures of the samples were measured by powder X-ray diffraction (PXRD) using a Bruker D8 Advance X diffractometer (Cu Kα: λ = 1.5418 Å). Thermogravimetric analyses (TGA) were conducted on a DTG-60 Shimadzu thermal analyst system under a heating rate of 10^°^C/min and a nitrogen flow rate of 50 cm^3^/min. Fourier transform infrared (FTIR) spectra were obtained on a Bruker VERTEX 70. Scanning electron microscope (SEM) images were acquired using a Hitachi SU4800-II cFEG SEM at an accelerating voltage of 2.0 kV. Ultraviolet/visible (UV/Vis) and fluorescence spectra were recorded on a Jasco V-750 spectrophotometer and Edinburgh FLS980, respectively. The absolute photoluminescence quantum yield was obtained using an Edinburgh FLS980 fluorescence spectrophotometer equipped with an integrating sphere. For fluorescence decay measurements, picosecond pulsed light-emitting diode (EPLED-380, wavelength: 377 nm, pulse width: 947.7 ps; EPLED-295, wavelength: 300 nm, pulse width: 833.7 ps) were used. Phosphorescence spectra were obtained using an Edinburgh FLS980 fluorescence spectrophotometer at 77 K with a 10 ms delay time after excitation using a microsecond flash lamp. The microsecond flash lamp produces short, typically a few μs, and high irradiance optical pulses for phosphorescence decay measurements ranging from microseconds to seconds. The kinetic measurements, afterglow spectra and lifetimes were also measured using an Edinburgh FLS980 fluorescence spectrophotometer. Excitation-phosphorescence mapping was measured using Hitachi F-4700 with a 25 ms delay time under ambient conditions. The lifetime (τ) of the luminescence were obtained by fitting the decay curve with singl-exponential or multi-exponential decay functions.

## 3 Results and discussion

**FIGURE 1:**
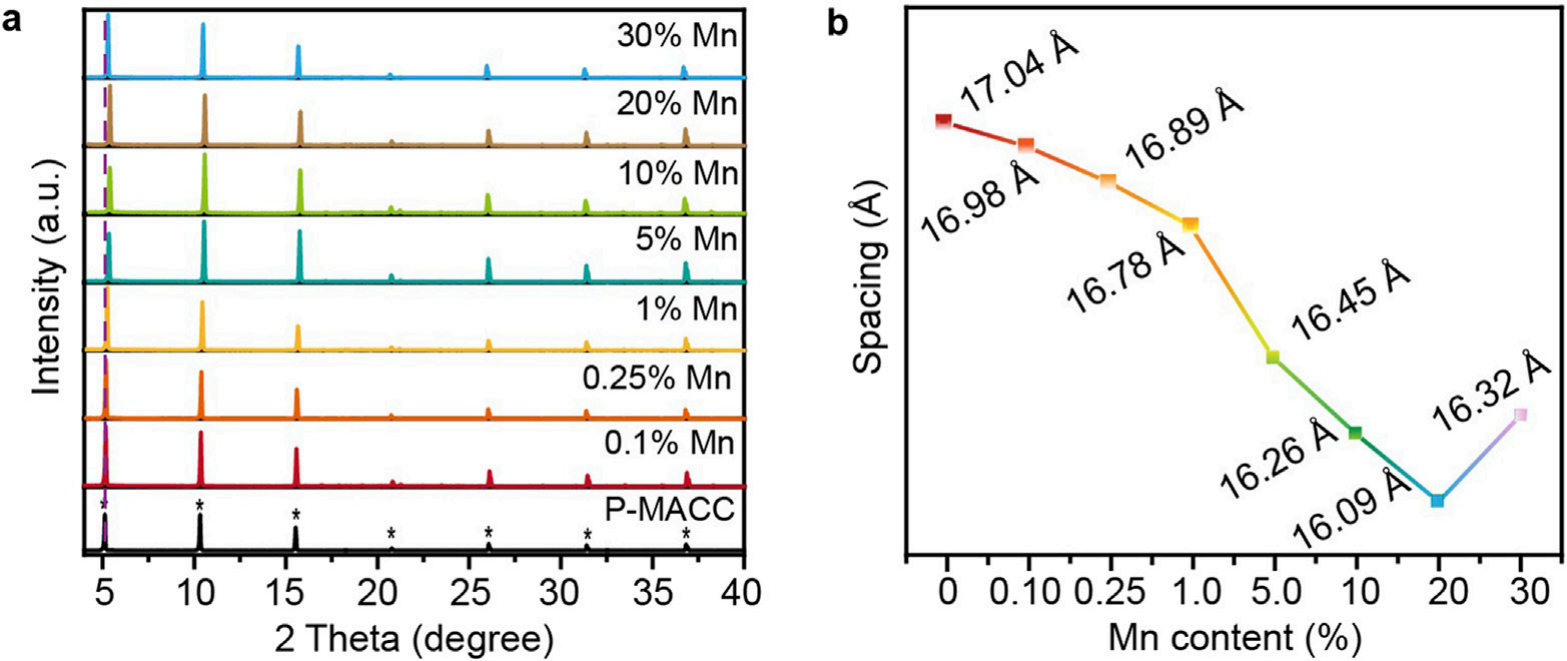
**(a)** Powder XRD patterns of P-MACC:x%Mn and **(b)** their corresponding interlayer spacing parameters.

Powder X-ray diffraction analysis was conducted on as-prepared halide perovskites P-MACC and its Mn^2+^-doped counterparts to determine whether the doping process affects the materials’ phase structure. As shown in Figure 1A, the PXRD patterns indicate that P-MACC:x%Mn exhibits PXRD characteristics similar to those of P-MACC, with strong periodic diffraction peaks along the (00L) planes. No significant diffraction peaks from other crystal planes were observed, further confirming that P-MACC:x%Mn shares the same space group as P-MACC, and the original material’s structure remains intact. Moreover, P-MACC:x%Mn retains the preferred orientation along the c-axis, which favors the formation of a layered structure with alternating inorganic and organic layers (Smith et al., 2019). Besides, the sharp diffraction peaks and increased diffraction intensity of the (00L) planes suggest that Mn^2+^ doping enhances the material’s crystallinity.

2D hybrid halide perovskite materials are always characterized by a layered structure, typically described by the interlayer spacing parameter (Mao et al., 2019). The interlayer spacing d refers to the distance between two adjacent inorganic layers in the material’s structure. This spacing can be calculated using the data from the first diffraction peak in the PXRD pattern and Bragg’s equation (2dsinθ = nλ, where θ denotes the diffraction angle, λ stands for the X-ray wavelength, and n refers to the diffraction order). As shown in Figure 1B, within the doping range of 0.1%–20% Mn^2+^, the interlayer spacing shows a slight decreasing trend. This decrease is attributed to the smaller ionic radius of Mn^2+^ compared to Cd^2+^, leading to lattice contraction and thus a shift of the diffraction peak at 2θ ≈ 5° towards higher angles (Liu et al., 2018a; Liu et al., 2018b; Liu et al., 2018c; Liu Z. et al., 2017). However, when the Mn^2+^ doping concentration reaches 30%, the first diffraction peak exhibits an anomalous shift toward lower angles. This phenomenon may be due to that excess Mn^2+^ not only substitutes Cd^2+^ in the inorganic layers but also occupies interlayer spaces, leading to an increase in the interlayer spacing.

The positions of absorption peaks in Fourier-transform infrared (FTIR) spectroscopy can be used to identify specific functional groups and chemical bonds within a material, allowing for further investigation into the internal structure and vibrational modes of organic components before and after Mn^2+^ doping. As illustrated in Supplementary Figure S4, both P-MACC and P-MACC:20% Mn exhibit N-H vibrational peaks corresponding to the amino group (-NH_2_), consistent with the characteristic vibrational peaks of the reactants' chemical structures. The N-H absorption peak of–NH_2_ in P-MACC appears around 3,066 cm^-1^, and in P-MACC:20% Mn, it shifts slightly to around 3,064 cm^-1^, indicating no significant peak position displacement. Furthermore, a comparison of the FTIR spectra reveals that the shapes, intensities, and positions of other absorption peaks remain remarkably similar, suggesting that the incorporation of Mn^2+^ at a certain concentration does not significantly affect the infrared vibrations of organic amines in the compounds. Notably, the FTIR spectrum of the P-MACC perovskite shows a prominent characteristic vibrational peak around 3,500 cm^-1^, which is likely attributed to the hydroxyl (-OH) peak from water in the air.

**FIGURE 2:**
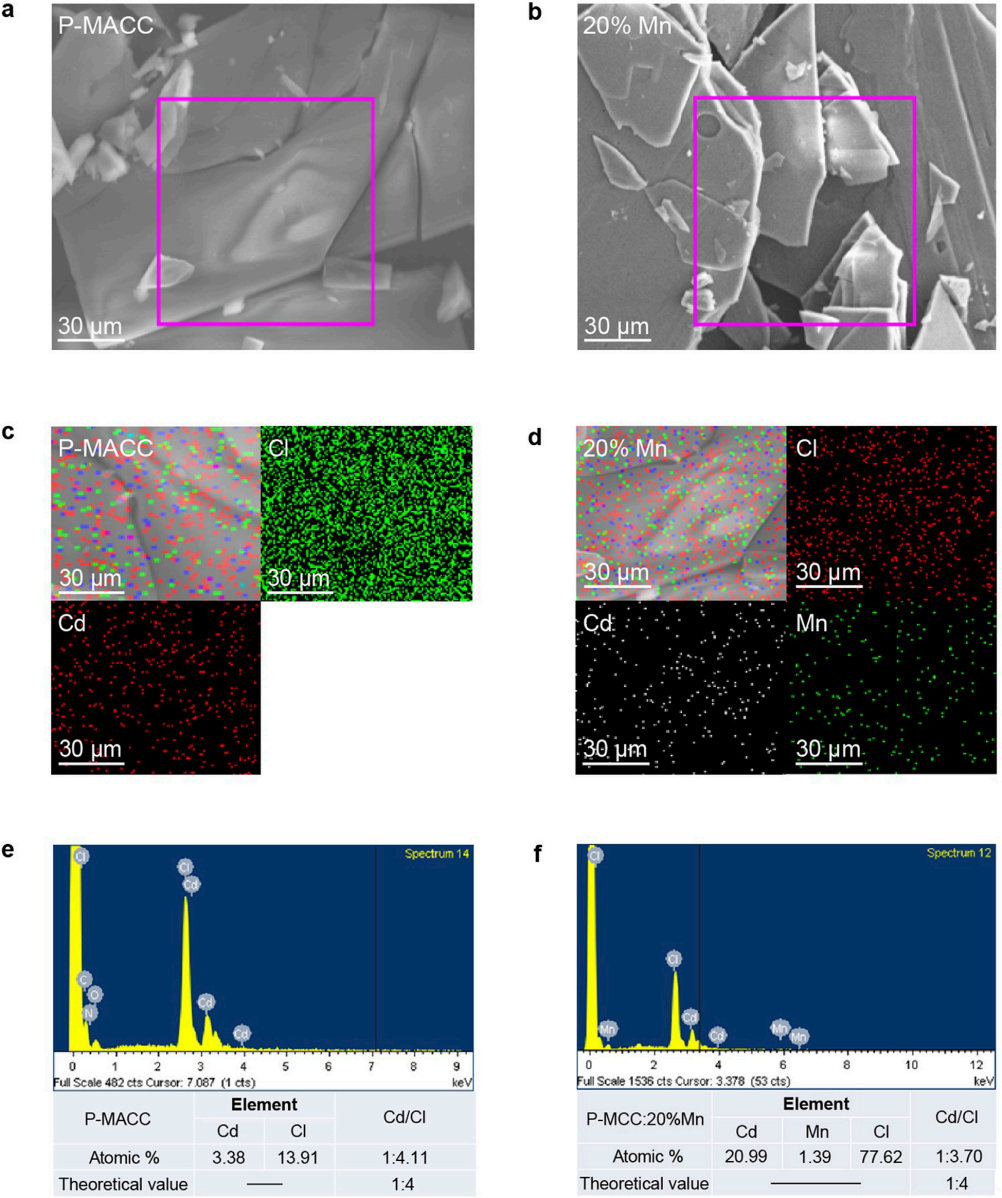
SEM images of **(a)** P-MACC and **(b)** P-MACC:x%Mn. Element mappings results of **(c)** P-MACC and **(d)** P-MACC:x%Mn. Energy-dispersive X-ray spectroscopy (EDS) results of **(e)** P-MACC and **(f)** P-MACC:x%Mn.

Scanning electron microscopy (SEM) can be used to observe the microstructure of materials, providing insight into their morphological characteristics. SEM analysis was conducted on the 2Dhybrid halide perovskites P-MACC and P-MACC:20% Mn. As shown in Figures 2A, B, both P-MACC and P-MACC:20% Mn exhibit a layered structure formed by the stacking of sheet-like growths. This is attributed to the influence of larger organic ligands in 2D perovskites, which results in a more pronounced layered morphology compared to three-dimensional halide perovskites (Castelli et al., 2019). Additionally, this indicates that the material retains the layered characteristics of 2D perovskites after Mn^2+^ doping. Elemental mapping can further reveal the distribution of elements within the materials. As shown in Figures 2C, D, both P-MACC and P-MACC:20% Mn contain Cd and Cl elements, while P-MACC:20% Mn also includes Mn, with all elements uniformly distributed throughout both materials. Figures 2E, F display the energy-dispersive X-ray spectroscopy (EDS) spectra for P-MACC and P-MACC:20% Mn, respectively. The comparison confirms the presence of Mn^2+^ in the P-MACC:20% Mn, although at a relatively low doping level (1.39%), which is consistent with Inductively coupled plasma mass spectrometry (ICP-MS) result (1.51%). This finding further demonstrates the successful incorporation of Mn^2+^ into P-MACC host. Additionally, the actual molar ratios of Cd to Cl in both materials are approximately 1:4.11 and 1:3.70, respectively, which are close to the theoretical molar ratio of 1:4 for 2D perovskites, confirming that both materials exhibit the characteristics of 2D structure and the chemical formula should be (P-MA)_2_(Cd_1-x_Mn_x_)Cl_4_.

To further confirm the successful incorporation of Mn^2+^ into P-MACC host, we conducted electron paramagnetic resonance (EPR) spectroscopy analysis. Supplementary Figure S5 displays the EPR spectrum of P-MACC:20% Mn, revealing a sextet hyperfine coupling pattern characteristic of the unpaired 3 d^5^ electron spin (S = 5/2) interacting with the nuclear spin (I = 5/2) (Mantel et al., 2004). The average hyperfine coupling constant for P-MACC:20% Mn is 9.38 mT (93.8 G), which is comparable to the hyperfine coupling constant observed in Mn-doped L_2_PbX_4_ systems. As a result, the sextet hyperfine splitting confirms the presence of Mn^2+^ within P-MACC crystal lattice.

Thermal stability is a crucial property of halide perovskites, with higher thermal stability generally indicating superior performance. To investigate the changes in thermal stability before and after Mn^2+^ doping, thermogravimetric analysis (TGA) was conducted on P-MACC and P-MACC:20% Mn over a temperature range of 25°C–800°C. As shown in Supplementary Figure S6, the initial thermal decomposition temperature of P-MACC:20% Mn is 284°C, which is 17°C higher than that of P-MACC, indicating that the introduction of Mn^2+^ enhances the thermal stability of P-MACC. In addition, both P-MACC and P-MACC:20%Mn exhibit two distinct decomposition stages. For P-MACC, the first stage begins at 267°C with a mass loss of approximately 32%, attributed to the volatilization of organic components. The second stage starts at 432°C, with a mass loss of around 42%, primarily due to the volatilization of inorganic CdCl_2_. In contrast, the first decomposition stage for P-MACC:20% Mn begins at 284°C with a mass loss of about 39%, also due to the volatilization of organic molecules. Notably, P-MACC:20% Mn shows a minor mass loss of approximately 1.5% before reaching 284°C, likely due to the desorption of a small amount of solvent or moisture. The second decomposition stage begins at 470°C, with a mass loss close to 32%, similarly attributed to the volatilization of inorganic CdCl_2_.

**FIGURE 3:**
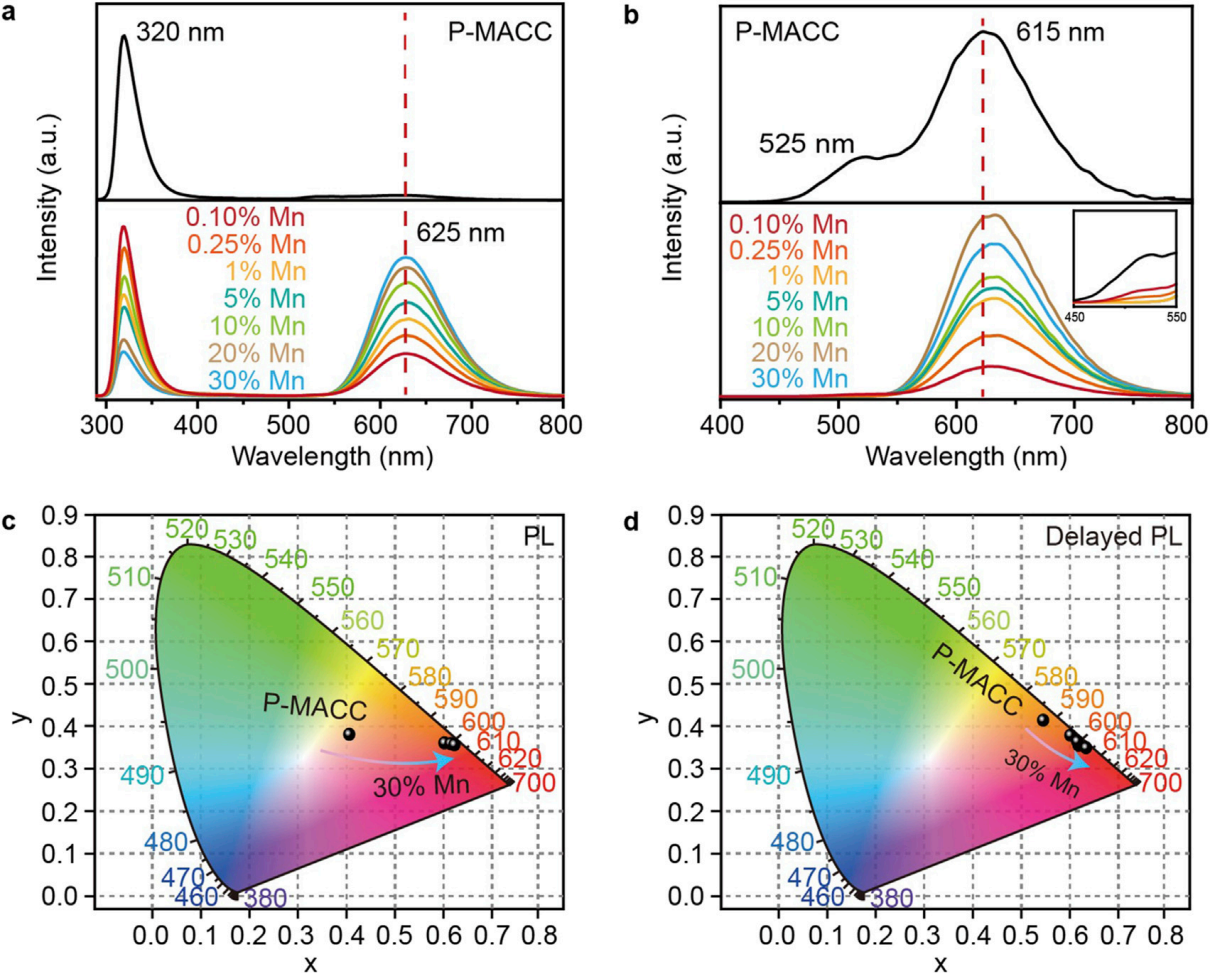
**(a)** Fluorescence and **(b)** phosphorescence spectra of P-MACC and P-MACC:x%Mn and **(c, d)** corresponding CIE coordinate diagrams.

To investigate the impact of Mn^2+^ doping on the luminescent properties of the materials, we characterized their optical properties using fluorescence and phosphorescence spectroscopy. Figure 3A shows the fluorescence spectra of the pristine and Mn^2+^-doped 2Dhybrid halide perovskites. For a fair comparison of intensity, all samples were prepared with equal amounts of powder and excited at a wavelength of 270 nm. The emission spectrum of pure P-MACC exhibits a sharp emission peak at 320 nm, accompanied by a weak broad emission band at longer wavelengths. In contrast, P-MACC:x%Mn materials display a strong broad emission centered at 625 nm. Although there is also an emission peak at 320 nm, its intensity decreases with increasing Mn^2+^ doping, while the emission intensity at 625 nm gradually intensifies. Figure 3B presents the phosphorescence spectra of the materials. The pristine P-MACC shows dual emission peaks at 525 nm and 615 nm. However, after Mn^2+^ doping, the peak at 525 nm gradually disappears, as shown in the inset of Figure 3B, and the spectrum shifts to a broad emission with a dominant peak centered at 625 nm. We speculate that the energy at 525 nm is being transferred to the 625 nm peak, thereby achieving higher emission efficiency.

To gain a clearer understanding of the materials’ color characteristics, we processed the fluorescence and phosphorescence spectra data using the Commission Internationale de l’Eclairage (CIE) standard color measurement system. As shown in Figures 3C, D, the fluorescence color coordinate of P-MACC is (0.41, 0.39), placing the color in the orange-yellow region. With the introduction of Mn^2+^ doping, the color coordinates shift to around (0.61, 0.37), moving the color towards the red region. For phosphorescence, the color coordinate of P-MACC is (0.54, 0.42), and Mn^2+^ doping shifts the coordinate to approximately (0.61, 0.36). Overall, both the fluorescence and phosphorescence color regions of MACC:x%Mn are predominantly orange-yellow, but with increasing Mn^2+^ doping, the color shifts progressively towards red. Aforementioned results can be visually confirmed by the photographs of P-MACC:*x*% Mn powders with varying Mn^2+^ doping concentrations under ambient and UV light (Supplementary Figure S7).

**FIGURE 4:**
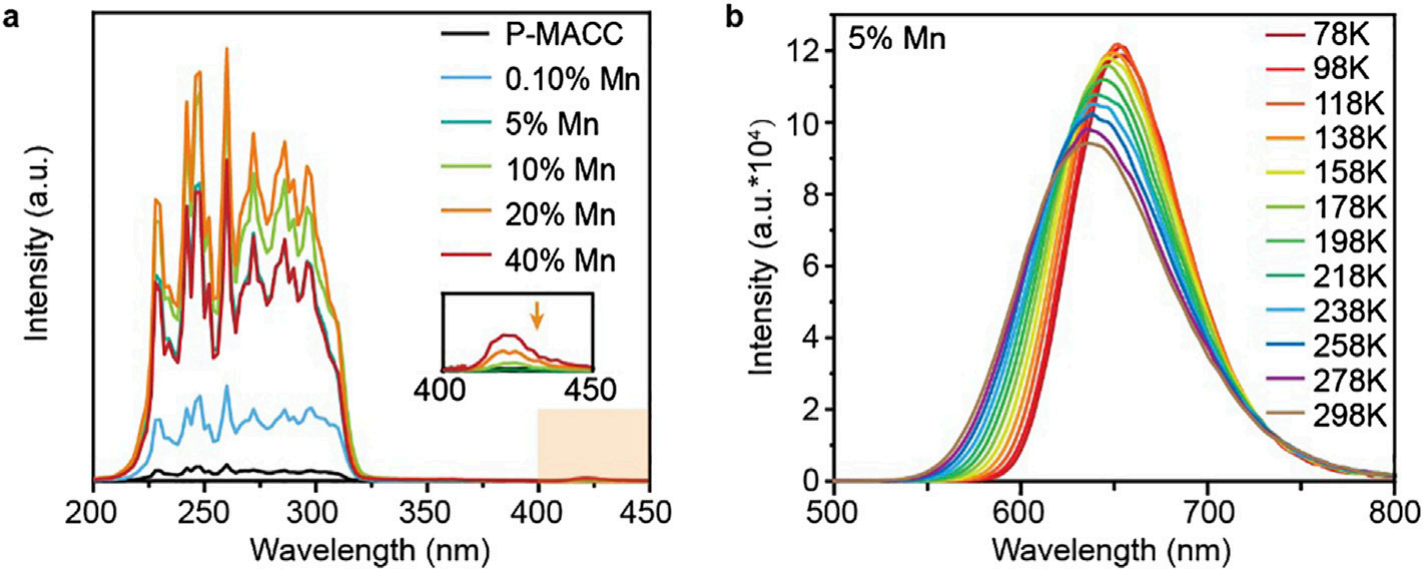
**(a)** Photoluminescence excitation spectra of P-MACC:x%Mn and **(b)** the temperature-dependent emission spectra of P-MACC:5%Mn.

To confirm that the 625 nm emission peak is attributable to the internal energy level transitions of Mn^2+^, we conducted relevant characterizations on both pristine P-MACC and Mn^2+^-doped counterparts. Figure 4A presents the photoluminescence excitation (PLE) spectra of P-MACC with varying Mn^2+^ doping concentrations. The sharp exciton peaks in the 200 nm–350 nm range are attributed to the absorption of the host excitons. Interestingly, MACC:x%Mn samples show additional characteristic peaks in the 400 nm–450 nm range, which correlate positively with the Mn^2+^ concentration. This result suggests that in the doped system, not all absorption peaks arise from the host excitons, but intermediate bandgap states also emerge with Mn^2+^ doping. The relatively low intensity of these intermediate bandgap states indicates that the emission energy of Mn^2+^ primarily originates from the absorption of the host excitons. To further understand the emission behavior of Mn^2+^ within P-MACC host, we examined the temperature-dependent emission spectra of P-MACC:5% Mn over a range of 78 K–298 K. As shown in Figure 4B, as the temperature increases from 78 K to 298 K, the emission band of MACC:5% Mn shows a blue shift from 655 nm to 625 nm. This shift is due to the thermal expansion of the host lattice, which reduces the d-d orbital splitting of Mn^2+^, consequently widening the ^4^T_1_→^6^A_1_ bandgap (Zhang et al., 2020; Yuan et al., 2017). This behavior is similar to the characteristics observed in Mn^2+^-doped ZnS and ZnSe nanocrystals (Yuan et al., 2014).

**FIGURE 5:**
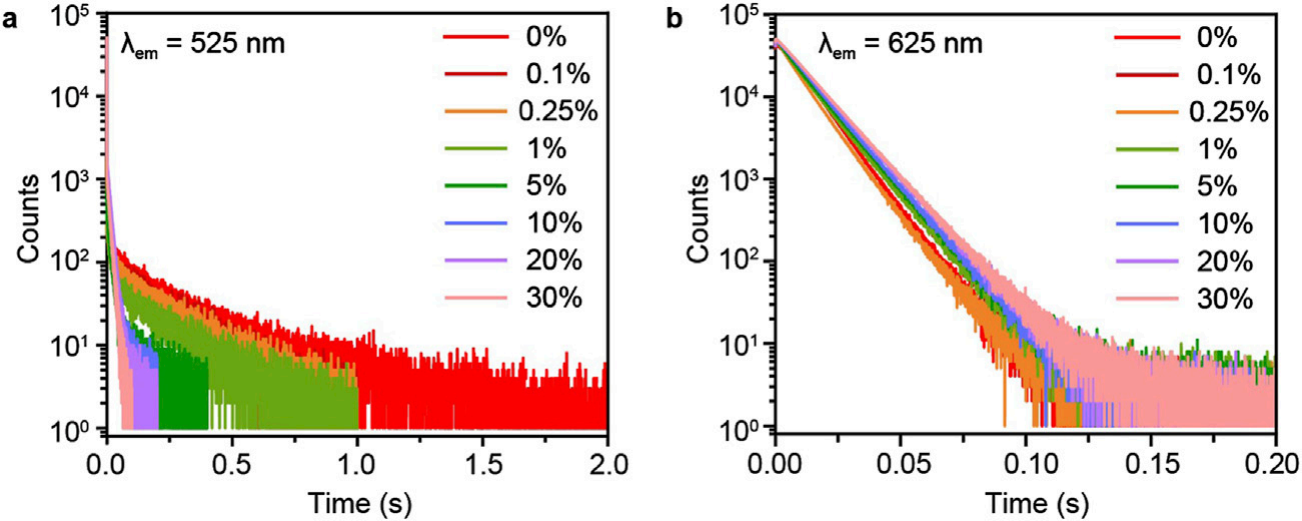
Time-resolved decay curves of P-MACC:x%Mn monitored at emission wavelength of **(a)** 525 nm and **(b)** 625 nm.

To verify the energy transfer between P-MACC host Mn^2+^ dopant, we conducted time-resolved spectroscopy measurements on the phosphorescence peaks of the materials before and after Mn^2+^ doping. As shown in Figure 5, with initially increasing doping concentration of Mn^2+^, the emission lifetime of P-MACC host at 525 nm gradually quenches, while the lifetime at 625 nm shows a slight increase. However, as the doping concentration further increases, the lifetime at 625 nm remains nearly unchanged. The slight increase in lifetime at 625 nm may be due to some degree of energy transfer from the excitons at the nearby energy level. Since the energy at 615 nm is relatively low, the increase in Mn^2+^ concentration does not significantly extend the lifetime at 625 nm. Combined the energy quenching of P-MACC host and increased luminescence intensity of Mn^2+^, it is evident that the energy at 525 nm is utilized to enhance the overall luminescence of the materials.

To gain a clearer understanding of the spectral peak lifetimes before and after Mn^2+^ doping, we conducted time-resolved emission spectroscopy (TRES) characterizations for P-MACC and P-MACC:20%Mn, as shown in Supplementary Figure S8. Results reveal that, as time progresses, the emission intensity gradually decreases for both samples. Under the same delay conditions, P-MACC:20% Mn exhibits the strongest emission intensity in the range of 560 nm–660 nm, while P-MACC shows the strongest emission intensity in the 450 nm–600 nm range. This result indicates that Mn^2+^ doping alters the peak position of the strongest emission intensity. Additionally, the emission intensity of P-MACC and P-MACC:20%Mn reach their approximately three orders of magnitude at 0.75 s and 1.5 s, respectively. We also collected the time-resolved decay curves of P-MACC:x%Mn monitored at 320nm, 525 nm and 625 nm, respectively, as shown in Figure 5 and Supplementary Figure S9. We found that the fluorescence lifetimes are nearly consistent (∼1.99 ns). We hypothesize that there is a large energy gap between the fluorescence emission peak of the P-MACC host and the emission of Mn^2+^ ions, which may make energy transfer difficult (Supplementary Figure S9). Clearly, P-MACC has a longer lifetime across the entire spectral range than P-MACC:20% Mn. Specifically, the lifetime at the 525 nm peak is significantly longer in P-MACC compared to P-MACC:20%Mn, further confirming that the energy at 525 nm decreases after Mn^2+^ doping. Therefore, we can calculate the energy transfer efficiency (Φ) using the following equation Φ = 1- τ/τ_0_ where τ, τ_0_ are the lifetime monitored at 525 nm in the presence and absence of Mn^2+^ ions, respectively. The Φ reaches 76% for P-MACC:20%Mn suggesting that a large proportion of triplet excitons has been transferred to the excited states of Mn^2+^ ions.

**FIGURE 6:**
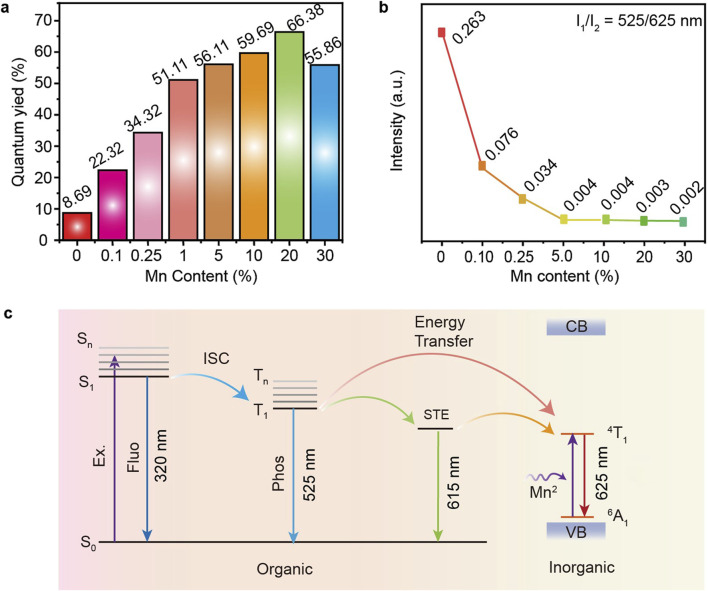
**(a)** The measured PLQYs for P-MACC:x%Mn. **(b)** The PL emission intensity ratio of wavelength monitored at 525 nm and 625 nm. **(c)** Possible energy transfer and emission mechanism in P-MACC:x%Mn.

To further validate the hypothesis that the energy at 525 nm is utilized to enhance the material’s luminescence, we conducted PLQY characterizations on P-MACC and P-MACC:20% Mn samples. Figure 6A illustrates the PLQY of the materials as a function of Mn^2+^ doping concentration. As the doping percentage rises from 0.1% to 20%, PLQY increases from 8.69% to 66.38%, an improvement of nearly 8 times. This enhancement is attributed to the introduction of new energy levels by Mn^2+^ and effective energy transfer, thereby enabling efficient Mn^2+^ emission (Liu H. et al., 2017). However, when the doping percentage exceeds 20%, excessive Mn^2+^ leads to the degraded quality of host crystal lattice and enhanced Mn^2+^-Mn^2+^ interactions, causing luminescence quenching (Yuan et al., 2017). Figure 6B shows the relationship between the relative luminescence intensity at 525 nm and 625 nm and the Mn^2+^ content after spectral normalization. As the Mn^2+^ content increases, the relative intensity declines, indicating that the energy at 525 nm is transferred to Mn^2+^, which in turn induces efficient phosphorescence emission. Based on the above discussion, emission mechanism for the high-efficiency luminescence of P-MACC:x%Mn is proposed, as depicted in Figure 6C. Under UV excitation, the system is excited to the S_1_ state, which can undergo intersystem crossing to the T_1_ state, followed by a transition to the self-trapping exciton (STE) state, resulting in corresponding energy level emission. Mn^2+^ doping introduces a new energy level (internal transition ^4^T_1_→^6^A_1_), enabling energy transfer from STE state to ^4^T_1_ state, or from the T_1_ state to ^4^T_1_ state, thereby facilitating the ^4^T_1_→^6^A_1_ radiative recombination. The high-efficiency luminescence of the Mn^2+^-doped materials is attributed to two factors: 1) Mn^2+^ doping enables efficient utilization of energy; 2) Mn^2+^ provides an effective pathway for the recombination of ^4^T_1_ excited state to ^6^A_1_ ground state. As a result, the PLQY of P-MACC:x%Mn is remarkably improved compared to pristine P-MACC.

**FIGURE 7:**
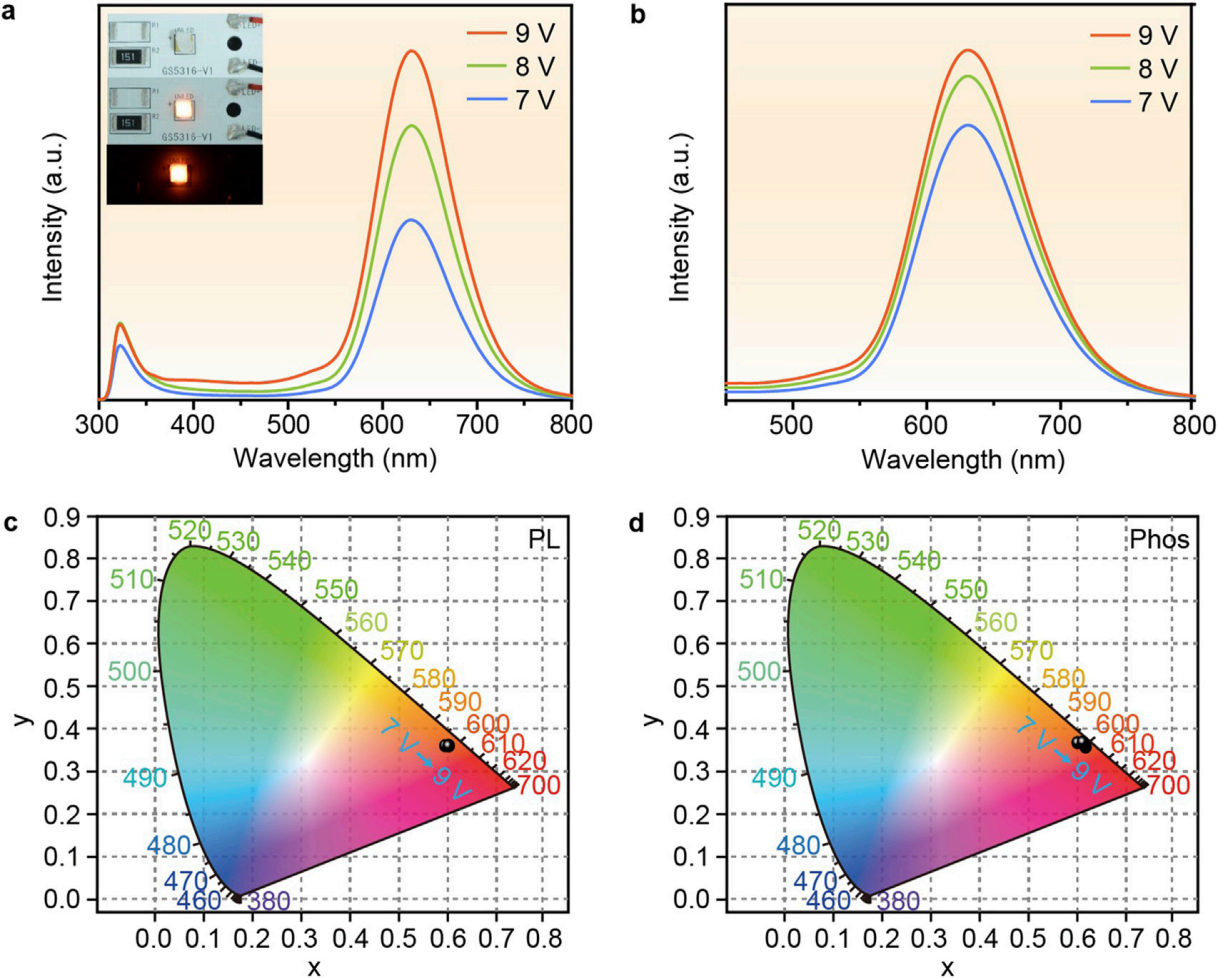
**(a)** The fluorescence and **(b)** phosphorescence spectra of LEDs based on P-MACC:20% Mn under different driving voltage and **(c, d)** corresponding CIE coordinate diagrams. Insets **(a)** are the device images under daylight with applied voltage on and off under day light, and the device illuminated in darkness.

Based on the excellent luminescent properties and thermal stability of P-MACC:x%Mn, we demonstrate their lighting applications. P-MACC:20% Mn samples are selected to directly coat onto LEDs chips, and the devices are driven with varying voltages to achieve emission, followed by spectral testing. Figure 7A shows the fluorescence spectra under different voltage drives, with the inset displaying photos of the fabricated LED devices. As the voltage increases, the emission intensity increases accordingly. Figure 7B presents the phosphorescence spectra under different driving voltage, that higher working voltage leads to stronger phosphorescence. Figures 7C, D indicate that the color coordinates of both fluorescence and phosphorescence remain relatively stable with varying voltages, demonstrating the materials’ excellent voltage stability.

## 4 Conclusion

In this work, a series of 2D hybrid halide perovskite P-MACC:x%Mn were synthesized by partially substituting Cd^2+^ with Mn^2+^, achieving efficient red RTP emission. Structural characterizations revealed that the successful incorporation of Mn^2+^ did not alter the original 2D perovskite layer structure. The P-MACC:x%Mn exhibited strong red phosphorescence emission at 625 nm, attributed to the ^4^T_1_→^6^A_1_ energy transition of Mn^2+^. In addition, the introduction of Mn^2+^ significantly enhanced the luminescence efficiency, with the PLQY increasing from 8.69% to 66.38%, representing an 8.0-fold improvement. As the Mn^2+^ doping concentration increased, the lifetime at 525 nm gradually decreased, which is resulted from that Mn^2+^ doping introduced new luminescent energy levels and enabled efficient energy transfer from host to Mn^2+^. Based on the superior optical properties of P-MACC:20% Mn, the material was coated onto LEDs to create red-emitting devices. This study demonstrates that Mn^2+^ plays a crucial role in improving the luminescence efficiency of 2D hybrid halide perovskites, providing a pathway for achieving efficient RTP in 2D hybrid halide perovskite systems.

## Data Availability

The original contributions presented in the study are included in the article/Supplementary Material, further inquiries can be directed to the corresponding authors.
