# Supplementary material for: Achieving efficient red room-temperature phosphorescence in two-dimensional hybrid halide perovskites by manganese doping
Source: Front Chem. 2025 Mar 21;13:1533513. doi: 10.3389/fchem.2025.1533513 (PMC11968679; doi:10.3389/fchem.2025.1533513)
Supplement: Supplementary file 1 [file DataSheet1.pdf]

Supplementary Material

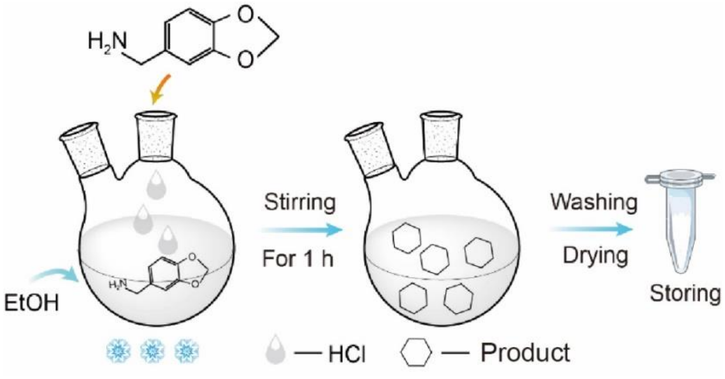

Figure S1. The synthesis process of preparing organic amines salts in this work.

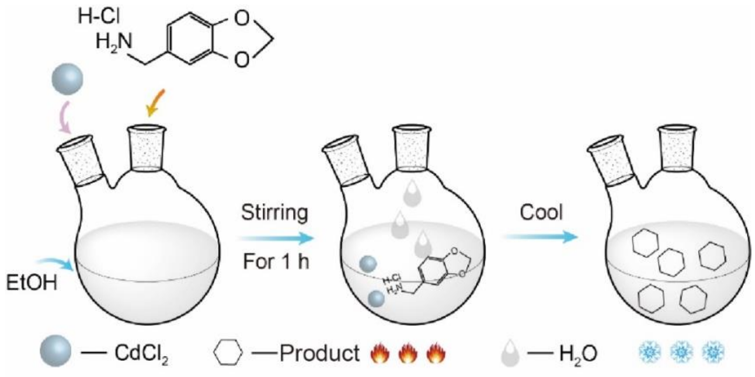

Figure S2. The synthesis process of preparing 2D halide perovskites without Mn^{2+} doping in this work.

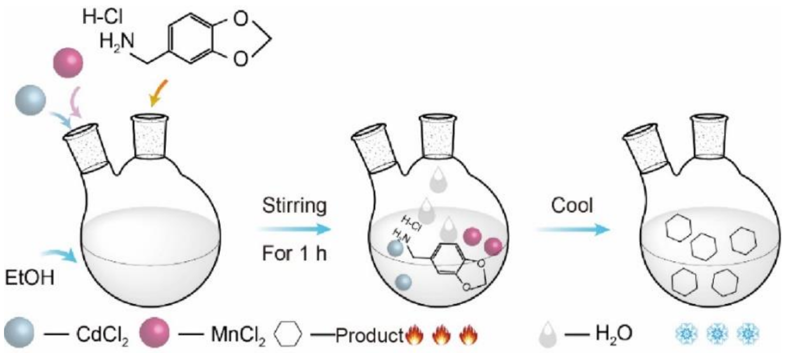

Figure S3. The synthesis process of preparing 2D halide perovskites with Mn^{2+} doping in this work.

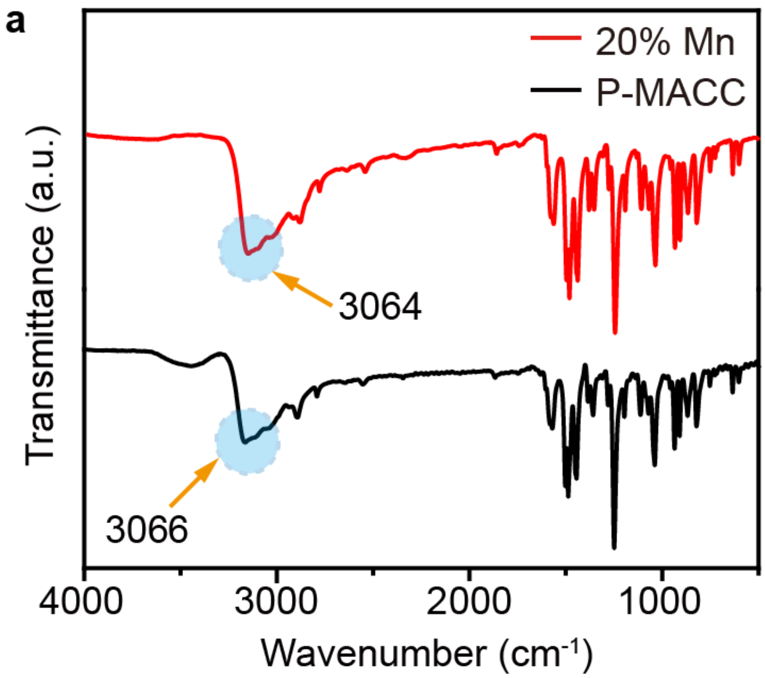

Figure S4. Fourier-transform infrared patterns of 2D halide perovskites before and after Mn^{2+} doping in this work.

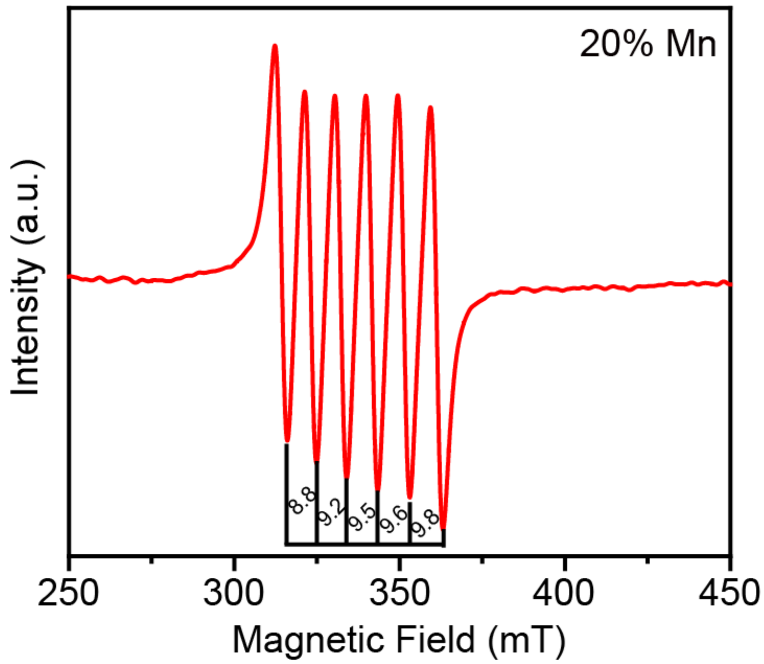

Figure S5. Electron paramagnetic resonance spectrum of P-MACC:20% Mn.

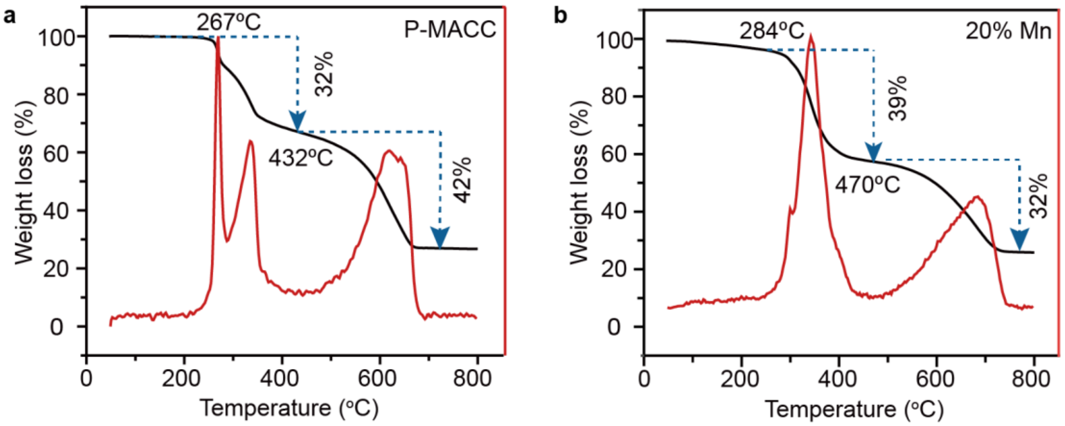

Figure S6. Thermogravimetry (black) and derivative thermogravimetry (red) curves of **(a)** P-MACC and **(b)** P-MACC:20% Mn.

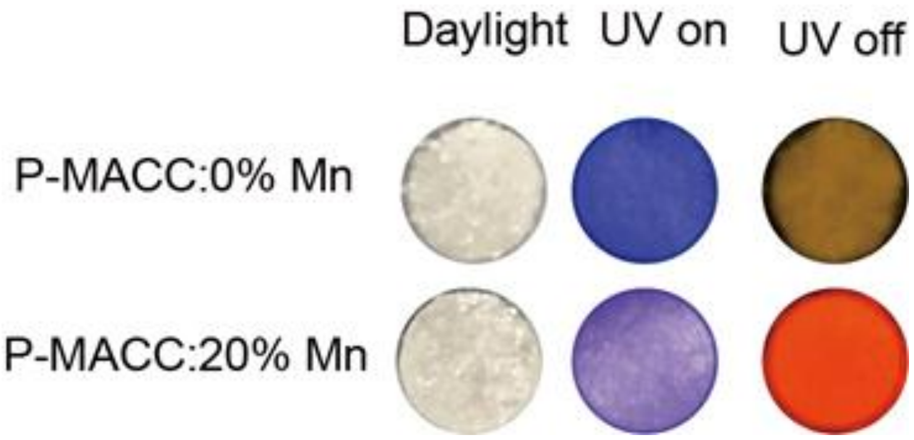

Figure S7. photographs of P-MACC:x% Mn powders with varying Mn^{2+} doping concentrations under ambient and UV light.

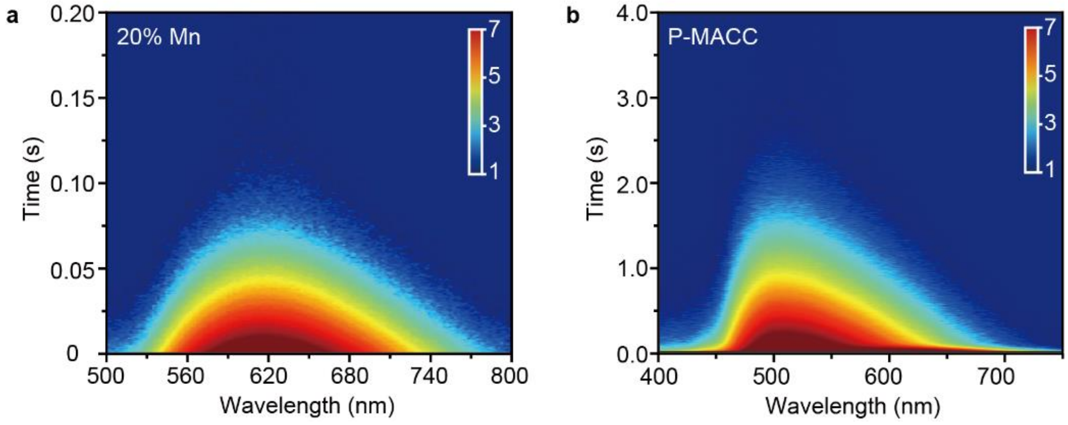

Figure S8. Time-resolved emission spectroscopy results of **(a)** P-MACC:20% Mn and **(b)** P-MACC.

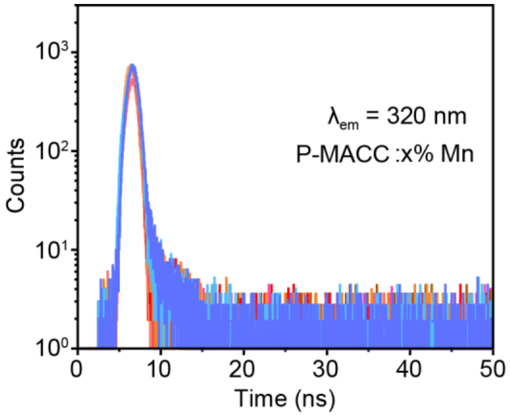

Figure S9. Time-resolved decay curves of P-MACC:x%Mn monitored at 320 nm.
